# Multi-phase deep learning model for automated disease classification from cardiac cine MRI

**DOI:** 10.1098/rsif.2025.0303

**Published:** 2025-10-15

**Authors:** Nicharee Srikijkasemwat, Mauricio Villarroel, Abhirup Banerjee

**Affiliations:** ^1^ Department of Engineering Science, University of Oxford, Oxford, Oxfordshire, UK; ^2^ Division of Cardiovascular Medicine, University of Oxford, Oxford, Oxfordshire, UK

**Keywords:** cardiovascular disease, cine MRI, classification, deep learning, explainability

## Abstract

Cardiovascular diseases (CVDs) are the major cause of death worldwide. Magnetic resonance imaging (MRI) is the gold standard modality for CVD diagnosis because of its ability to distinguish different types of soft tissues without the use of ionizing radiation. Cine MRI allows us to see the contractile function of the heart, and it is a safe method for patients with chronic kidney diseases. The aim of this work was to develop a deep learning model for automated classification of common CVDs from cine MRI while providing the model explainability. We investigated single-phase models based on either the end-diastolic (ED) or end-systolic (ES) phase using seven baseline deep learning models including ResNet, DenseNet and VGG. We then developed a multi-phase model including both ED and ES phases to incorporate cardiac function for CVD classification. While the single-phase model for the ED and ES phases yielded the highest test F1-scores of 71.0% and 76.0% respectively, the multi-phase model achieved a test F1-score of 77.0%. To better understand the model performance, we used explainability to visualize regions of the heart that exhibit characteristics of each disease. Our work has demonstrated that deep learning models can automatically and effectively classify CVDs from cine MRI while justifying classification, thus building trust from the clinical community.

## 1. Introduction

Despite significant advancements in medical treatment, cardiovascular diseases (CVDs) resulted in approximately 20 million deaths worldwide in 2021 [1], which is equivalent to 55 000 deaths per day on average. Diagnosing CVDs can be a difficult task as the symptoms can be similar in different patients. Cardiac function plays a vital role in diagnosing the disease, as well as in patient management, risk evaluation and making therapy decisions. Among various methods to analyse cardiac functions, electrocardiography (ECG), echocardiography, magnetic resonance imaging (MRI), computed tomography (CT) and single photon emission computed tomography (SPECT) are the most popular [2,3].

Cardiac MRI is considered the gold standard imaging modality due to its ability to distinguish different types of soft tissues [2,4]. It can detect both structural and functional abnormalities in the heart [4]. The two most common cardiac MRI modalities are late gadolinium enhancement (LGE) and cine MRI [5]. LGE MRI is effective in detecting myocardial lesions and fibrosis, which are found in various CVDs. However, it requires intravenous injection of gadolinium-based contrast agent, which can be dangerous in people who are allergic to the contrast agent and patients with severe kidney failure as their kidneys may struggle to dispose of the contrast agent [6,7]. On the other hand, cine MRI does not require a contrast agent and acquires images at different cardiac phases, allowing us to visualize the heart structure, motion and contractile function [5,6].

To evaluate cardiac function from cine MRI for CVD diagnosis, clinicians need to measure several parameters from the images. These include ejection fraction of left and right ventricles, stroke volume, left ventricular mass and myocardial thickness. Accurate measurements require precise epicardial and endocardial boundary delineations and identification of end-diastolic (ED) and end-systolic (ES) frames. However, the quantification process tends to vary with observers, which can result in significant variations in measured cardiac parameters. Moreover, the process can be time-consuming [2,8].

Artificial intelligence (AI) can assist clinicians in diagnosing and speeding up the analysis. Several attempts have been made to perform automated disease diagnosis using machine learning techniques based on derived cardiac parameters [2]. Since the measurement process of cardiac parameters is not only time-consuming but also tends to vary between observers, the use of deep learning could replace the manual delineation and metric calculation. This would allow us to analyse the cardiac images directly, thus accelerating the diagnosis process. In this work, we focus solely on the classification of CVDs directly from cine MRI, without requiring segmentation or manual feature extraction.

Since cine MRI does not necessitate the administration of contrast agents, it is an effective imaging technique for patients with CVDs who also have chronic kidney failure. Therefore, our study aimed to use deep learning models to classify common CVDs, namely, myocardial infarction (MI) with altered left ventricular ejection fraction, dilated cardiomyopathy (DCM), hypertrophic cardiomyopathy (HCM), abnormal right ventricle (ARV) and subjects without any cardiac disease (NOR) directly from cine MR images and provide the reason for the model decision using model explainability.

### 1.1. Related works

Several studies have explored the use of machine learning and deep learning approaches for cardiac disease classification using cardiac MRI. As summarized in the review article by Martin-Isla *et al.* [3], most methods rely on cardiac parameters—such as ventricular volumes and ejection fraction—typically obtained after segmentation of cardiac structures; some also incorporate clinical variables (e.g. genetic data, age, sex). These parameters can vary between subjects, and their assessment may be subjective. In addition, traditional machine-learning pipelines require explicit feature selection, so important information may be missed. By contrast, our work performs direct image-based classification from cine MRI, bypassing both handcrafted features and segmentation.

Bernard *et al.* [2] reported that machine learning techniques, such as using a support vector machine or random forest on handcrafted features extracted from segmentation, could classify CVDs with high accuracy. They reported an accuracy of 96% for CVD classification, achieved by Khened *et al.* [9], employing random forest after extracting features from the segmentation using Dense U-Net.

Deep learning methods, on the other hand, can perform complex tasks without explicit feature selection [10]. Therefore, using deep learning to analyse images directly may provide more accurate results and a better understanding of the CVDs. Snaauw *et al.* [11] proposed a method combining DenseNet and U-net architectures to perform cardiac segmentation and classification on four CVD classes and a healthy class. The authors hypothesized that segmentation has a regularizing effect on feature learning for classification and accordingly designed a loss function based on multi-task learning, taking both segmentation and classification into account. The network consists of three distinct branches: main, segmentation and diagnosis. The main and diagnosis branches form a DenseNet architecture, while the main and segmentation branches form a U-net-like structure. The inputs to this network were six consecutive slices of the ES phase, ED phase and the subtraction volume of these two phases. Their method achieved 78% accuracy in the joint segmentation-classification setting, and 68% accuracy when performing classification alone—notably lower than the handcrafted feature-based approach reported by Bernard *et al.* [2].

Germain *et al.* [12] used existing deep learning models to classify DCM, HCM and healthy subjects from cine MRI. The models, including VGG-16, ResNet-50V2, InceptionResNetV2 and DenseNet-201, were pre-trained on the ImageNet dataset, fine-tuned on the last layers and finally passed to fully connected layers to output three classes. Additionally, the authors used a model named VGG-concat, consisting of two pre-trained VGG-16 models, one for the diastolic frame and the other for the systolic frame input. The feature maps of two VGG-16 models were concatenated and passed to the fully connected layers. For the base models, the InceptionResNetV2 and VGG-16 were the best performers, with the test accuracy of VGG-16 for diastolic and systolic inputs as 0.961±0.011 and 0.952±0.012, respectively. The VGG-concat with both diastolic and systolic inputs outperformed them with a test accuracy of 0.982±0.009.

### 1.2. Contributions

Our main contributions are summarized as follows:

— We used deep learning models to classify CVDs directly from cardiac cine MRIs without relying on handcrafted features, using only the ED and ES phases—two clinically important time points that capture the structural and functional extremes of the cardiac cycle.
— We performed a systematic evaluation of seven state-of-the-art deep learning models for CVD classification on the Automatic Cardiac Diagnosis Challenge (ACDC) dataset, based on single-phase inputs (ED and ES).
— We proposed a lightweight multi-phase model that combines the learnt features from both ED and ES phases, achieving improved classification performance by capturing dynamic cardiac function.
— We employed Smooth Grad-CAM++ to highlight the cardiac regions influencing model predictions, supporting model explainability and clinical trust.

The rest of the article is structured as follows: §2 describes the dataset used in this study, data preprocessing, data augmentation and the single-phase and multi-phase models for CVD classification. The results are presented in §3 and discussed in §4. The article concludes in §5.

## 2. Material and methods

### 2.1. Dataset

We used the ACDC dataset [2], containing cine MRI of 150 subjects from five equally distributed classes, namely, MI, DCM, HCM, ARV and NOR. The ARV class was determined based on the criterion of arrhythmogenic right ventricular cardiomyopathy/dysplasia (ARVC/D) [2,13]. An expert provided the ED and ES frames according to the motion of the mitral valve. The 150 subjects were divided into 100 subjects in the training set, evenly with 20 subjects in each class, and the remaining 50 in the test set, with 10 subjects per class. We selected the mid cavity slice of MRI at the ED and ES frames for CVD classification, since they contain the most information.

We split the images in the training dataset into training and validation sets using stratified k-fold cross-validation. Using k=4, we obtained four sets, of which three sets are training sets and one set is a validation set, with each set containing 25 subjects. The stratified sampling in cross-validation divided the subjects from each class equally, resulting in balanced representation in each set; that is, out of 25 subjects in each set, there were five subjects from each class.

### 2.2. Data preprocessing

The MR images in the ACDC dataset contain other organs apart from the heart. Hence, cropping is needed to remove irrelevant information as well as to retain the centre of the heart as the centre of the image. To determine the cropping size, we considered two main factors: first, the maximum width and height of the hearts in the dataset and second, the effect of rotation, which would be applied later as part of the augmentation process.

**Figure 1.**
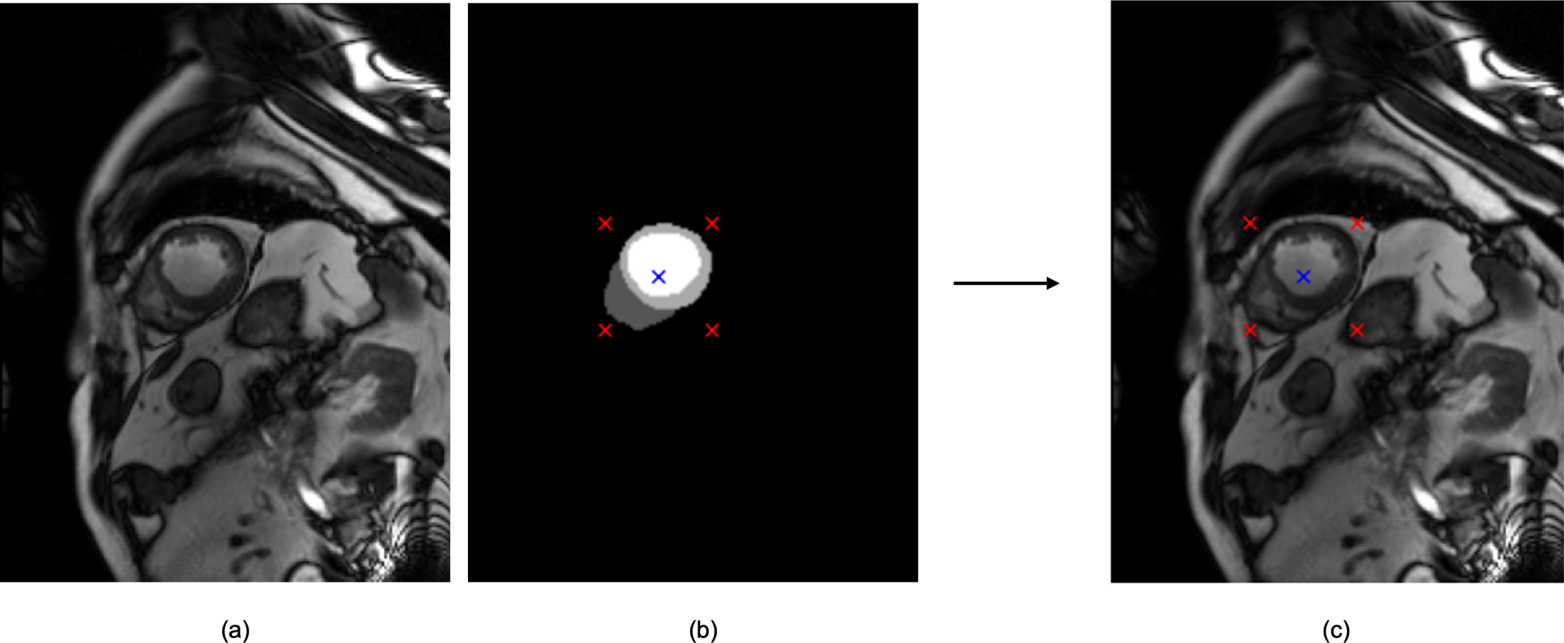
An example of cine MRI (a) and corresponding segmentation mask (b) of a subject with myocardial infarction in the ACDC dataset. The segmentation mask allowed the computation of the centre of the heart (as a blue cross), width and height (as a red cross) (c).

First, in order to determine the cropping size and position, the information about the centre of each heart, its width and height is required. We identified the region of the heart using the ground-truth segmentation mask, each containing the left ventricular (LV) cavity, LV myocardium, right ventricular (RV) cavity and the background. To find the location of the heart, we identified all foreground pixels of the LV cavity, LV myocardium and RV cavity and computed the minimum and maximum values of these pixel locations to find the height, width and centre of each heart. No segmentation information is supplied to the classification models; all training is image-based. Figure 1 shows an example of an MR image, corresponding segmentation mask, along with the centre of the heart (as a blue cross) and its height and width (as a red cross).

**Figure 2.**
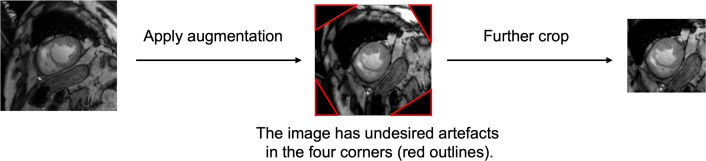
The MR images were first cropped to be 1.5 times larger than the maximum heart size. After stratified k-fold cross-validation, augmentation techniques, including rotation, adjusting brightness and contrast, were applied to the images in the training set. The undesired artefacts in the four corners (shown by red outlines) were removed by further cropping the images before training the model.

Before cropping, it is essential to consider the effect of rotation, which would be applied to training images during the augmentation process. Rotation can cause undesirable artefacts at the four corners (shown by red outlines in figure 2). To ensure they were not included, we first cropped the images to be 1.5 times larger than the maximum size, with the centre of the heart being at the centre of the images. Then, after applying the augmentations, we further cropped them. This factor of 1.5 was calculated based on the maximum rotation angle in the augmentation process. Since we applied random rotation ranging between −30° and +30° to the training images, the maximum angle of rotation was 30°. Hence, in order to avoid the artefacts at the corners, the images needed to be 1.5 times larger than the maximum heart size.

After applying the cropping strategy, two datasets were created: one for the ED frames, and the other for the ES frames. Each dataset consisted of 100 images in the training set and 50 images in the test set. There were five evenly distributed classes in each training and test set, with 20 images per class in the training set and 10 images per class in the test set.

### 2.3. Data augmentation

Data augmentation was applied on-the-fly to the training images to increase variability and improve model generalizability. We applied random brightness and contrast adjustments using the ColorJitter transformation from the PyTorch torchvision library [14]. The brightness factor was sampled uniformly from [0.5, 1.5]. This scales the intensity of each pixel, resulting in darker (50%) or brighter (150%) versions of the image. The transformation is defined as


(2.1) $$p_{\text{out}}=p_{\text{in}}\times \alpha ,$$

where α∈[0.5,1.5] and $p_{\text{in}},p_{\text{out}}$ denote the input and output pixel values, respectively. The contrast factor was sampled uniformly from [0.5, 1.5], modifying pixel values relative to the image mean. This is expressed as


(2.2) $$\begin{array}{lcr} & p_{\text{out}}=(p_{\text{in}}-\mu )\times \beta +\mu , & \end{array}$$

where β∈[0.5,1.5] and μ is the mean pixel intensity of the image. Higher contrast factors increase visual contrast by expanding the intensity range around the mean. Saturation was not applied, as it is relevant only to RGB images and has no effect on greyscale cine MRI.

In addition, we applied random rotation between −30° and +30° to increase the variety of the images. After applying augmentation to the training images, both training and validation images were cropped at the centre of the image (as described in detail in §2.2). All images in the training, validation and test datasets were then resized to 128×128 pixels, converted into tensors and applied normalization using the mean and standard deviation of the ImageNet dataset [15]. Although normalization is applied, it does not cancel the effects of brightness and contrast augmentation. Normalization is a fixed affine transformation,


(2.3) $$\begin{array}{lcr} & p_{\text{norm}}=\frac{p_{\text{aug}}-\mu_{\text{norm}}}{\sigma_{\text{norm}}}, & \end{array}$$

where ‘$p_{\text{aug}}$’ denotes pixel values after applying data augmentation and ‘$p_{\text{norm}}$’ denotes pixel values after normalisation using fixed constants ‘$\mu_{\text{norm}}$’ and ‘$\sigma_{\text{norm}}$’.

### 2.4. Single-phase models for cardiovascular disease classification

**Figure 3.**
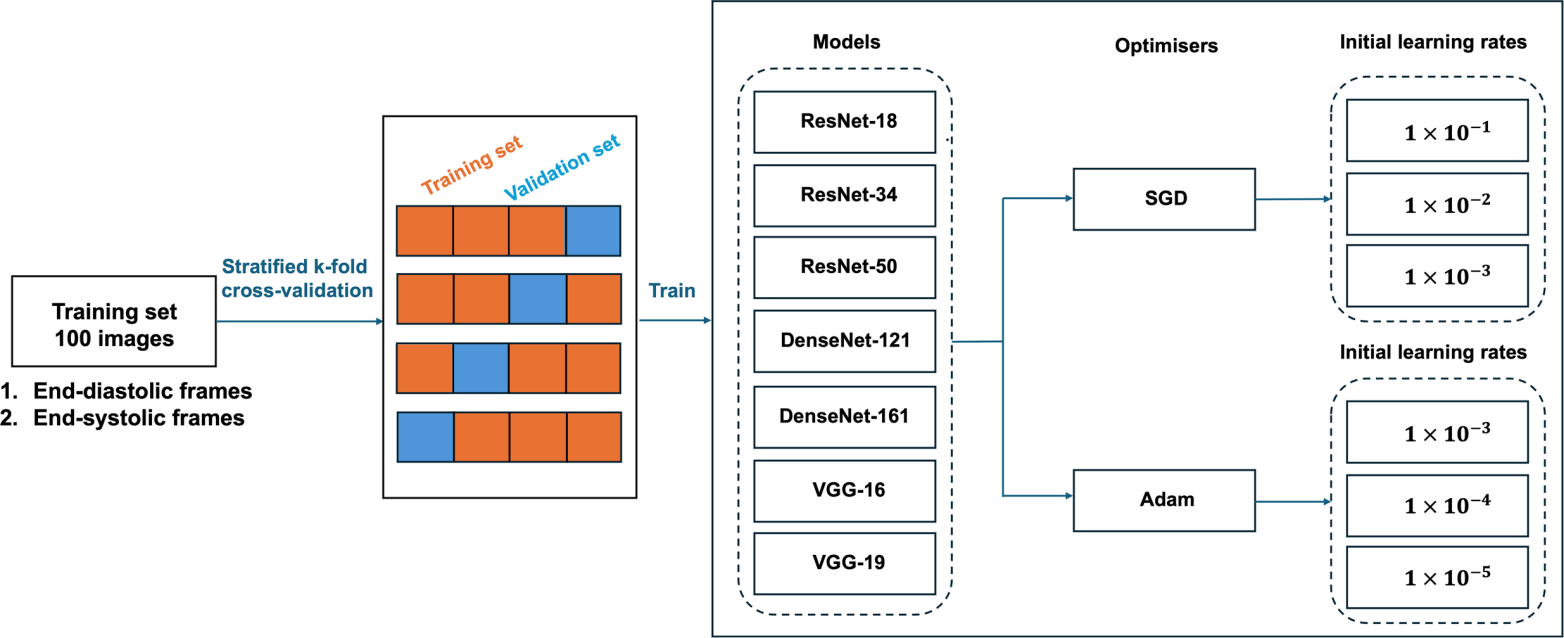
The overall training process of the single-phase model. The images were first split into training and validation sets using stratified k-fold cross-validation. Each fold was trained with different combinations of deep learning models, optimizers and initial learning rates.

The training process was carried out systematically in order to compare various deep learning models with different optimizers and initial learning rates. Figure 3 shows the overall training process.

We trained each fold using different combinations of deep learning architectures, optimizers and initial learning rates. We used seven deep learning architectures, two optimizers and three different initial learning rates for each optimizer, resulting in 42 combinations in total. So, each deep learning model was trained with six combinations of optimizers and initial learning rates in order to select the best hyperparameters for each model. For each fold, the weights of the models at the epoch with the highest validation accuracy were saved.

We trained each model using cross-entropy loss for 150 epochs, with a batch size of 20. The learning rates were decreased by a factor of 10 every 20 epochs. The following list provides information on the deep learning models, optimizers and initial learning rates used in our experiment:

— Seven deep learning architectures: ResNet-18, ResNet-34, ResNet-50, DenseNet-121, DenseNet-161, VGG-16 and VGG-19. These models were pre-trained on the ImageNet dataset [15]. We initialized the networks with the pre-trained weights and applied fine-tuning to all layers, allowing the models to adapt to the domain-specific features of cardiac cine MRI. The last fully connected layer was adjusted to output one of the five classes: MI, DCM, HCM, ARV and NOR.
— Two optimizers: stochastic gradient descent (SGD) and Adam.
— Three initial learning rates were used with each type of optimizer. For SGD, the learning rates were $1\times 10^{-1}$, $1\times 10^{-2}$ and $1\times 10^{-3}$. For the Adam optimizer, the initial learning rates were $1\times 10^{-3}$, $1\times 10^{-4}$ and $1\times 10^{-5}$.

The average validation macro F1-score of each combination was computed across four folds. The combination with the highest average validation F1-score was selected to be the best single-phase model to form the multi-phase model described in §2.5. For this best single-phase model, we trained four different models on four folds. We selected the one with the highest validation F1-score and applied it on the held-out test dataset.

In order to examine the model’s explainability, we applied the Smooth Grad-CAM++ technique [16] to the last convolutional layers of these models and displayed the region that influenced the model’s decision.

### 2.5. Multi-phase models for cardiovascular disease classification

In this experiment, we considered both the ED and ES phases of the same subject together to incorporate the cardiac function information. We designed this multi-phase model by combining the two trained single-phase models: the best single-phase model for the ED phase and the best single-phase model for the ES phase, based on the highest average validation F1-scores.

**Figure 4.**
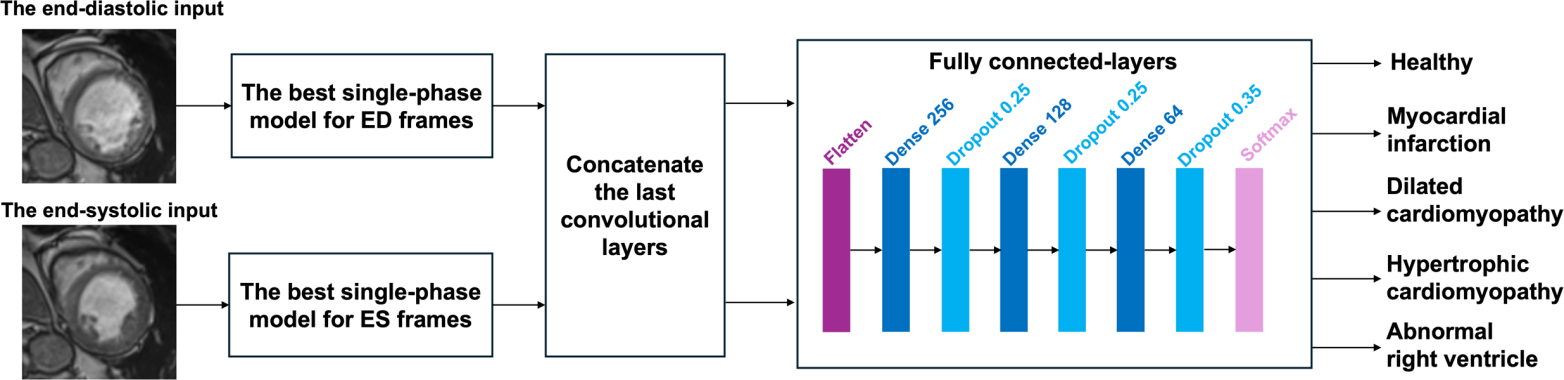
The multi-phase model considering both end-diastolic (ED) and end-systolic (ES) inputs together for multi-class CVD classification. The ED and ES inputs are passed through the single-phase models with the highest average validation F1-scores trained on the ED and ES phases, respectively. The last convolutional layers of the two models are concatenated and further trained with fully connected layers.

In the architecture of the multi-phase model, feature maps from the final convolutional layers of the ED and ES models were concatenated and passed through a fully connected (FC) block consisting of a Flatten layer, followed by fully connected layers with 256, 128 and 64 units, each followed by dropout layers with dropout rates of 0.25, 0.25 and 0.35, respectively. These values were selected based on preliminary experiments to balance regularization strength with model capacity. A final softmax activation layer was used to output the class probabilities for five disease categories. Figure 4 illustrates the full pipeline for the multi-phase model for multi-class CVD classification.

During training, only the FC layers were updated. The convolutional layers of the best-performing ED and ES single-phase models were kept frozen to reduce the number of trainable parameters and minimize the risk of overfitting. Additional regularization was achieved using dropout, on-the-fly data augmentation and stratified four-fold cross-validation.

We trained the multi-phase model for 150 epochs using cross-entropy loss. Similar to the single-phase models, here we also compared the performance of two optimizers: SGD and Adam. For SGD, the initial learning rates were $1\times 10^{-1}$, $1\times 10^{-2}$ and $1\times 10^{-3}$. For Adam, the initial learning rates were $1\times 10^{-3}$, $1\times 10^{-4}$ and $1\times 10^{-5}$. The learning rates were reduced by a factor of 10 every 20 epochs. We trained the multi-phase models based on the same four-fold stratified cross-validation splits as used in the single-phase model training, and evaluated the final model on the 50-subject held-out test set.

We also investigated the performance of multi-phase models on binary classification of each disease and healthy subjects. Since the ACDC dataset contains four disease classes and one healthy class, we considered four binary classification tasks: MI versus healthy, DCM versus healthy, HCM versus healthy and ARV versus healthy. We started by investigating the best-performing single-phase models for the ED and ES inputs in each binary classification task. In the case of multiple architectures achieving the same highest validation F1-scores, we chose the architecture with fewer parameters and a lower learning rate as these promote stable learning. Next, we combined these two models trained on ED and ES inputs to consider both phases together. In each case of binary classification, the input data consisted of 20 training subjects and 10 test subjects for each class. They were applied with the same cropping and augmentation techniques as the multi-class classification. We also retained the same set of optimizers and initial learning rates.

### 2.6. Statistical analysis

To assess the significance of performance differences between models, we compared the best-performing ED, ES and multi-phase models using bootstrap-based statistical testing. We generated 1000 bootstrap samples (*n* = 50, with replacement) from the original test set. Each of the three models was evaluated on the same set of bootstrap samples, ensuring that model comparisons were made on identical resampled distributions. For each bootstrap sample, we computed four evaluation metrics: F1-score, accuracy, sensitivity and specificity, and then calculated the mean and standard deviation of each metric across the 1000 samples.

We conducted one-sided paired *t*‐test, Wilcoxon signed-rank test and permutation test (10 000 resamples) on the bootstrap distributions for each metric across the following model pairs: ES versus ED, multi-phase versus ED and multi-phase versus ES [17–20]. These three pairwise comparisons, applied to four metrics, resulted in 12 statistical tests. Since we performed multiple comparisons, we applied a Bonferroni correction to control the family-wise error rate, adjusting the significance threshold to


(2.4) $$\begin{array}{lcr} & \alpha_{\text{corrected}}=\frac{0.05}{12}\approx 0.004. & \end{array}$$

A difference was considered statistically significant if the corresponding *p*-value was below this corrected threshold.

## 3. Results

### 3.1. Single-phase models for cardiovascular disease classification

The highest results of each deep learning model over two optimizers and three initial learning rates are presented in tables 1 and 2. The results are averaged across four folds. For the models trained on ED frames, the highest validation F1-score was 73.3±10.1%, achieved by ResNet-34 trained with Adam optimizer and an initial learning rate of $1\times 10^{-3}$. For the ES inputs, ResNet-50 trained with Adam with an initial learning rate of $1\times 10^{-3}$ yielded the highest validation F1-score of 82.8±4.7%. In addition to macro-averaged F1-score, we report macro-averaged sensitivity, specificity and accuracy across all classes. Macro-average means that these metrics were computed per class and averaged equally, which is appropriate given the class-balanced nature of the dataset. As a result, macro sensitivity and overall accuracy are numerically equivalent in this setting. We retain both metrics to maintain consistency with prior work and to support a complete interpretation of the model’s performance.

**Table 1.** Validation results over four folds of different deep learning architectures with the best optimizer and initial learning rate when trained on ED phases for multi-class classification. Bold values indicate the highest value for each metric.

architecture	settings	macro F1-score (%)	accuracy/macro sensitivity (%)	macro specificity (%)
ResNet-18	Adam, 1 × 10^−3^	68.3 ± 15.9	72.0 ± 12.6	93.0 ± 3.2
ResNet-34	Adam, 1 × 10^−3^	**73.3 ± 10.1**	**74.0 ± 9.5**	**93.5 ± 2.4**
ResNet-50	Adam, 1 × 10^−3^	71.5 ± 8.9	72.0 ± 8.6	93.0 ± 2.2
DenseNet-121	Adam, 1 × 10^−3^	71.3 ± 11.3	73.0 ± 8.2	93.3 ± 2.1
DenseNet-161	Adam, 1 × 10^−4^	72.5 ± 8.3	73.0 ± 8.9	93.3 ± 2.2
VGG-16	Adam, 1 × 10^−4^	67.3 ± 9.3	69.0 ± 8.2	92.3 ± 2.1
VGG-19	Adam, 1 × 10^−4^	61.5 ± 7.0	65.0 ± 5.0	91.3 ± 1.3

Reported quantities show mean ± standard deviation.

**Table 2.** Validation results over four folds of different deep learning architectures with the best optimizer and initial learning rate when trained on ES phases for multi-class classification. Bold values indicate the highest value for each metric.

architecture	settings	macro F1-score (%)	accuracy/macro sensitivity (%)	macro specificity (%)
ResNet-18	Adam, 1 × 10^−3^	78.8 ± 9.8	79.0 ± 9.5	94.8 ± 2.4
ResNet-34	Adam, 1 × 10^−3^	82.5 ± 4.7	82.0 ± 5.2	95.5 ± 1.3
ResNet-50	Adam, 1 × 10^−3^	**82.8 ± 4.7**	**83.0 ± 5.0**	**95.8 ± 1.3**
DenseNet-121	Adam, 1 × 10^−3^	78.3 ± 9.0	79.0 ± 7.6	94.8 ± 1.9
DenseNet-161	Adam, 1 × 10^−3^	78.8 ± 4.3	79.0 ± 3.8	94.8 ± 1.0
VGG-16	Adam, 1 × 10^−4^	70.8 ± 8.3	72.0 ± 8.6	93.0 ± 2.2
VGG-19	Adam, 1 × 10^−4^	74.8 ± 4.3	75.0 ± 3.8	93.8 ± 1.0

Reported quantities show mean ± standard deviation.

**Figure 5.**
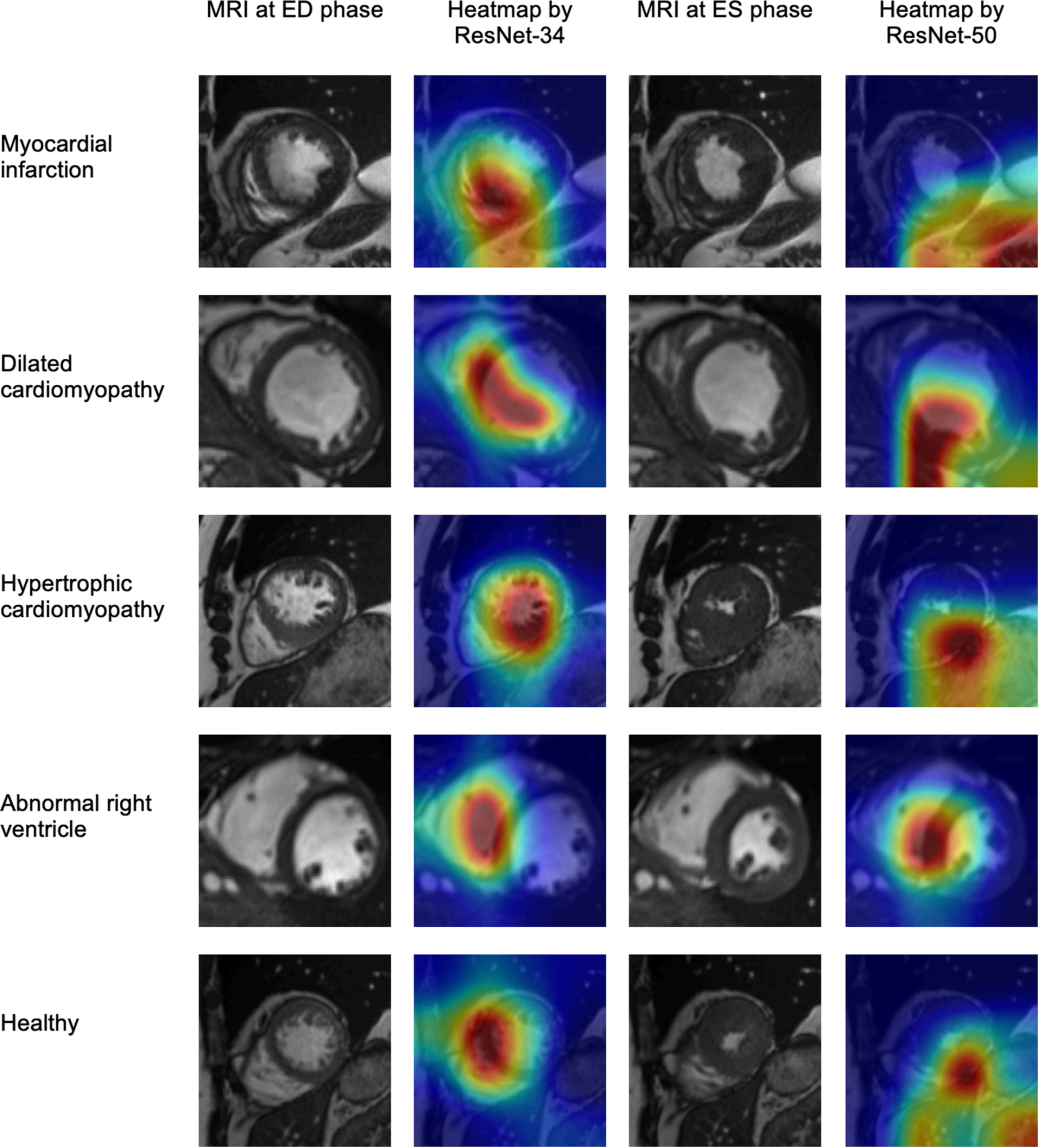
MR images at the end-diastolic (ED) and end-systolic (ES) phases, and the heatmaps showing the region that influences the model classification using Smooth Grad-CAM++. The first two columns denote the ED phase, whereas the last two columns denote the ES phase. Rows from top to bottom correspond to: MI, DCM, HCM, ARV and healthy subjects.

When testing the best single-phase models trained on the fold with the highest validation F1-score, the test F1-score reached 71.0% for the ED inputs and 76.0% for the ES inputs. The test accuracies were 72.0% and 76.0%, respectively. In addition, figure 5 shows the explainability results on the MR images of a subject from each group. The heatmaps were generated by applying Smooth Grad-CAM++ to the best single-phase models for the ED and ES phases, which were ResNet-34 and ResNet-50, respectively.

### 3.2. Multi-phase models for cardiovascular disease classification

Using both ED and ES phases and training further with the fully connected layers, the multi-phase model yielded the highest validation F1-score and accuracy of 87.0±3.8%. This was achieved using the SGD optimizer with an initial learning rate of $1\times 10^{-1}$ (table 3). The test F1-score and accuracy of this multi-phase model on the held-out test set were 77.0% and 78.0%, respectively.

**Table 3.** Validation results over four folds of the multi-phase model with different combinations of optimizers and initial learning rates. Bold values indicate the highest value for each metric.

settings	macro F1-score (%)	accuracy/macro sensitivity (%)	macro specificity (%)
SGD, 1 × 10^−1^	**87.0 ± 3.8**	**87.0 ± 3.8**	**96.8 ± 1.0**
SGD, 1 × 10^−2^	82.8 ± 4.2	84.0 ± 3.3	96.0 ± 0.8
SGD, 1 × 10^−3^	49.8 ± 12.9	57.0 ± 11.5	89.3 ± 2.9
Adam, 1 × 10^−3^	83.0 ± 3.8	83.0 ± 3.8	95.8 ± 1.0
Adam, 1 × 10^−4^	84.0 ± 5.7	84.0 ± 5.7	96.0 ± 1.4
Adam, 1 × 10^−5^	83.8 ± 6.6	84.0 ± 6.5	96.0 ± 1.6

Reported quantities show mean ± standard deviation.

**Figure 6.**
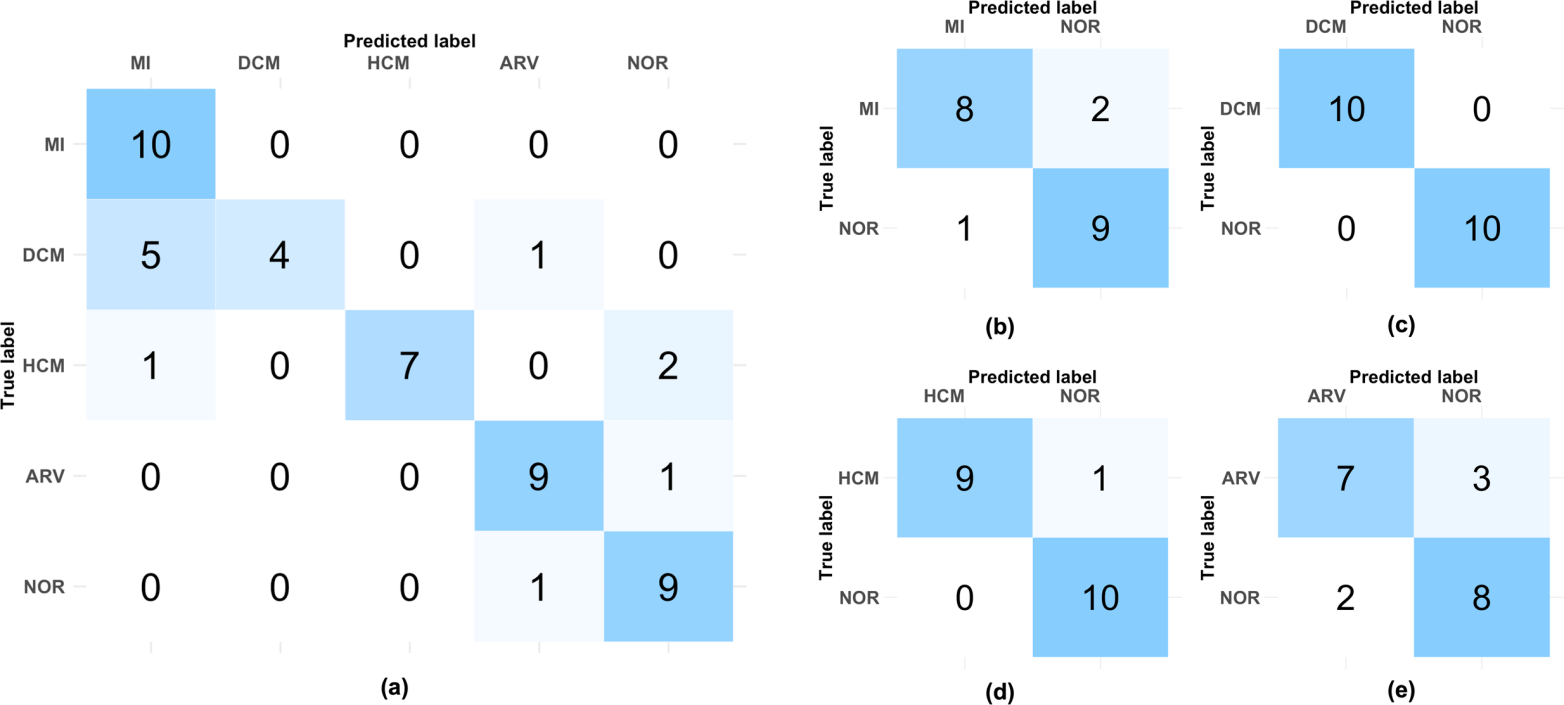
Confusion matrices of the multi-phase models on the test dataset for (a) multi-class classification and binary classifications of (b) MI versus NOR, (c) DCM versus NOR, (d) HCM versus NOR and (e) ARV versus NOR. MI: myocardial infarction; DCM: dilated cardiomyopathy; HCM: hypertrophic cardiomyopathy; ARV: abnormal right ventricle; NOR: healthy subjects.

We also applied the multi-phase model for binary classification. The confusion matrices of the multi-phase models for multi-class classification and binary classifications on the test dataset are presented in figure 6. Note that each of the binary classification models was tested on a total of 20 subjects (10 subjects from the disease class and 10 NOR subjects), whereas the multi-class model was tested on all 50 subjects in the held-out test dataset.

Among the four binary classification tasks, the highest result was achieved by the model between DCM and healthy subjects, achieving a perfect 100.0% test F1-score. For DCM classification, the best performing single-phase models for the ED and ES phases were both DenseNet-121 trained with the Adam optimizer and an initial learning rate of $1\times 10^{-3}$. For the ED frames, DenseNet-121 obtained a test F1-score of 85.0%, whereas for the ES frames, it yielded the perfect 100.0% test F1-score. The multi-phase model trained using the Adam optimizer with an initial learning rate of $1\times 10^{-4}$ achieved the highest average validation F1-score.

In the case of classifying subjects with HCM versus subjects without any known CVDs, the multi-phase model yielded a 95.0% test F1-score. This was achieved by using ResNet-50 and DenseNet-121 models with the Adam optimizer and an initial learning rate of $1\times 10^{-4}$. The ResNet-50 model achieved a test F1-score of 80.0%, trained with Adam and an initial learning rate of $1\times 10^{-3}$ on the ED phase. DenseNet-121 was trained with Adam and the same initial learning rate, achieving a test F1-score of 100.0% on the ES phase.

For MI detection, the multi-phase model achieved a test F1-score of 85.0%, using the SGD optimizer with an initial learning rate of $1\times 10^{-3}$. This multi-phase model was formed by two DenseNet-121 models, which performed best for both ED and ES phases. For the ED inputs, DenseNet-121 with Adam and an initial learning rate of $1\times 10^{-3}$ achieved 80.0% test F1-score. For the ES inputs, DenseNet-121 with the same configuration attained the 85.0% test F1-score.

Finally, for the classification between abnormal right ventricle and healthy subjects, the multi-phase model yielded 75.0% test F1-score. This multi-phase model consisted of DenseNet-161 and DenseNet-121, trained using the Adam optimizer with an initial learning rate of $1\times 10^{-5}$. The DenseNet-161 model achieved a test F1-score of 65.0% for the ED phase, whereas the DenseNet-121 yielded a test F1-score of 80.0%, when both models were trained with Adam and an initial learning rate of $1\times 10^{-3}$.

### 3.3. Statistical analysis

Table 4 reports the mean and standard deviation of the 1000 bootstrap samples, generated with replacement from the test set, for each of the three models. Based on these samples, all 12 pairwise comparisons (3 model pairs × 4 metrics) yielded statistically significant differences according to the Bonferroni-corrected significance level, i.e. all *p*-values were below the corrected significance level of 0.004 for all three statistical tests (one-tailed), namely paired *t*‐test, Wilcoxon signed-rank test and permutation test. This demonstrates that the multi-phase model significantly outperformed the best-performing ED and ES single-phase models across all four metrics; the ES model also significantly outperformed the ED model.

**Table 4.** Test results of the single-phase and multi-phase models for multi-class classification based on 1000 bootstrap samples. Bold values indicate the highest value for each metric.

model type	input data	architecture	settings	macro F1-score (%)	accuracy (%)	macro sensitivity (%)	macro specificity (%)
single-phase	ED	ResNet−34	Adam, 10^−3^	69.6 ± 6.8	71.9 ± 6.2	72.0 ± 5.8	93.0 ± 1.5
	ES	ResNet−50	Adam, 10^−3^	75.1 ± 6.2	76.0 ± 6.1	76.0 ± 6.2	94.0 ± 1.6
multi-phase	ED & ES	ResNet−34,	SGD, 10^−1^	**75.9 ± 6.1**	**78.0 ± 5.7**	**78.0 ± 5.2**	**94.5 ± 1.4**
		ResNet−50,					
		FC layers					

The bootstrap samples, which were drawn randomly with replacement from the 50 test subjects, were not perfectly class-balanced. This led to differences in accuracy and macro sensitivity values in table 4. Although the reported indices were quite similar, they were not numerically the same.

## 4. Discussion

This study introduces a modular deep learning framework that processes the ED and ES phases of cine MRI through separate, phase-specific deep learning models. Unlike prior approaches that combine multiple phases as a single input, our method enables tailored architecture selection for each phase and phase-specific explainability. We discuss both quantitative results and qualitative insights, and highlight how our design compares with established approaches.

### 4.1. Single-phase models for cardiovascular disease classification

Training the single-phase models on the ED phase yielded an overall validation F1-score ranging between 61.5% and 73.3%. For the ES phase, the validation F1-score was higher, ranging between 70.8% to 82.8%. Since the ES phase captures the heart’s contractile function, it enables a better assessment and, accordingly, the models can identify the pathology better as compared with the ED phase. This finding is further supported by the statistically significant differences observed between the ES and ED models, as confirmed by one-sided paired *t*-tests, Wilcoxon signed-rank tests and permutation tests across all evaluation metrics.

For the ED phase, ResNet-34 achieved the highest validation F1-score of 73.3±10.1%, followed by DenseNet-161, which yielded a high validation F1-score of 72.5±8.3%, only 1.1% lower than the ResNet-34. DenseNet-161 also attained 17.8% lower standard deviation, so it could be another good choice for CVD classification based on ED frames. For the ES phase, ResNet-50 achieved the top validation F1-score of 82.8±4.7%, closely followed by ResNet-34 (82.5±4.7%), with only 0.4% lower validation F1-score.

As noted in §3.1, macro sensitivity and overall accuracy are numerically equivalent in our setting due to the balanced class distribution. This equivalence simplifies interpretation while ensuring that per-class performance is adequately reflected in aggregate metrics.

Figure 5 demonstrates the explainability of the results. The first and third columns show the cine MRI at the ED and ES phases, respectively. The second column illustrates Smooth Grad-CAM++ visualizations for the ResNet-34 on the cine MRI at the ED phase, whereas the last column shows the same for the ResNet-50 at the ES phase. The red colour highlights the region of the MRI that influences model’s decision the most.

The first row of figure 5 shows the MRI of a patient with myocardial infarction. At the ED phase, the model focused on the left ventricular myocardium, potentially detecting the damaged area where there was a loss of myocardial tissue [21]. However, for the ES phase, the model underperformed as it focused on areas outside the left ventricle. For the patient with dilated cardiomyopathy (row 2 of figure 5), the models detected the thin wall in both phases, which is a characteristic of this disease [2,22]. At the third row, the heatmap of both ED and ES phases focused on the thickening muscle of the patient’s left ventricle, which is consistent with the characteristic of HCM [2,23]. For the patient with an abnormal right ventricle, the models focused on parts of the right ventricle at the ED phase (row 4). However, it did not consider the entire area of the enlarged right ventricle, which is the characteristic of this disease (dilation of the right ventricle) [23]. Finally, the last row shows the cine MRI of a healthy subject. Both heatmaps highlighted the myocardium of the left ventricle, though they highlighted at different locations.

In the challenge where the ACDC dataset was introduced, Bernard *et al.* [2] reported that most participants used traditional machine learning techniques to perform the classification task, including support vector machine and random forest [2,11]. Khened *et al.* [9] achieved the highest test accuracy of 96% using random forests. However, their traditional machine learning approach relied on handcrafted features, which could be subjective. The deep learning models applied in our work also provide explainability for the model decision by highlighting the important regions in the cine MR images, which is of particular significance in clinical settings. Importantly, the ACDC organizers noted that subjects with ambiguous clinical indices were excluded from the dataset [2], which may have created clearer diagnostic boundaries and probably contributed to the high reported accuracy of handcrafted feature-based methods [11].

### 4.2. Multi-phase models for cardiovascular disease classification

Considering both the ED and ES phases together, our proposed multi-phase model can learn different features of cardiac mechanics from different phases and become more effective at classifying CVDs. Our multi-phase model for five-class classification yielded a 77.0% test F1-score, higher than the F1-scores achieved by two single-phase models (ES: 76.0%, ED: 71.0%). Furthermore, the test results based on bootstrap sampling indicate that the performance of the multi-phase model is significantly better than that of the single-phase models (both ED and ES), as all pairwise comparisons yielded statistically significant results. This demonstrates that combining information from both ED and ES phases enables the model to more effectively learn discriminative features of the diseased heart.

In general, binary classifications yielded higher test F1-scores than multi-class classification. One reason for this might be the difference in dataset sizes. The model for the multi-class classification was tested on 50 subjects, whereas the models for binary classifications were tested on only 20 subjects. Although binary classifications achieved higher results, the model that recognizes more diseases is more practical in real life. Another possible reason might be that, due to the limited size of the dataset, it was difficult for the model to learn features to distinguish between five classes in multi-class classification. Trained on a larger dataset, the deep learning models are expected to perform better and generalize well for multi-class classification.

As observed in the confusion matrix in figure 6a, the multi-class classification model performed well in identifying MI, ARV and NOR subjects. However, it struggled to classify DCM and HCM subjects. The model misclassified 5 out of 10 DCM subjects as MI subjects. This could arise from the similar characteristics of DCM and MI, as some MI subjects can exhibit high left ventricular volume at the ED phase, as the heart compensates for the damaged area affected by MI [2].

When trained for the binary classification tasks, the model effectively learnt the features of DCM and HCM better than when trained on multi-class data, as visible in figure 6c,d. The characteristics of these diseases can be easily distinguished from those of healthy hearts. However, this distinction became more challenging when DCM and HCM were trained alongside MI. On the other hand, the model learnt the characteristics of MI and ARV and demonstrated better identification in the multi-class classification compared with the binary classification (figure 6b,e).

Snaauw *et al.* [11] proposed a multi-task learning framework that combines classification and segmentation using DenseNet and U-net architectures. Their classification-only model, which used a three-channel input comprising the ED phase, ES phase and their difference (ED−ES), achieved an accuracy of 68%. Their multi-task model improved performance to 78% accuracy. In contrast, our multi-phase model processes the ED and ES inputs through two independently trained models and fuses their learnt feature representations. This modular structure enables independent optimization of architectures for each phase, effective use of pre-trained backbones and phase-specific explainability via Smooth Grad-CAM++. Our model achieved 78.0% accuracy, representing a notable improvement over their classification-only approach. In future work, we aim to explore multi-task learning as a complementary strategy to further improve model performance.

A recent study by Liu *et al.* [24] applied a successive subspace learning (SSL) framework to the ACDC dataset for CVD classification, using three-dimensional deformation fields extracted from the ED and ES phases. The SSL method is lightweight and interpretable, computing weights in a feedforward manner without the need for backpropagation. It achieved up to 95% accuracy via five-fold cross-validation on 99 training subjects and outperformed a three-dimensional ResNet baseline (88% accuracy). While their results were not reported on the ACDC held-out test set and are therefore not directly comparable to ours, our multi-phase model achieved a similar validation accuracy of 87.0±3.8% using only two-dimensional image inputs and four-fold stratified cross-validation. Unlike SSL, which requires preprocessing to extract deformation fields, our model operates directly on raw cine MRI, offering a simpler and potentially more generalizable pipeline. SSL represents a promising direction that we intend to explore in future work.

Germain *et al.* [12] also incorporated the diastolic and systolic frames when classifying dilated and hypertrophic cardiomyopathy against healthy subjects based on a dataset of 1200 cine MRI from 534 subjects. The result was higher than that of models trained on a single-phase as input. They also suggested that including more frames could further improve the results. The experiments in our work have demonstrated that by including two frames, when the heart is maximally expanded (the ED phase) and when maximally contracted (the ES frame), there is more information for the model to learn, resulting in better diagnosis. The limited dataset size in our experiment may have restricted the learning of discriminative features in the multi-phase model. It is anticipated that the model would perform better when trained on a larger dataset. Additionally, incorporating additional phases in the cardiac cycle may further improve learning of the heart’s function, leading to better performance.

Zhang *et al.* [25] also found that using multiple phases results in a higher model performance than a single phase. The authors developed a deep learning model to detect MI from non-enhanced cardiac cine MRI. The study considered all 25 phases of cine MRI, as well as a single image at the ED phase. The model’s performance on the full 25-phase MRI was substantially better than that of the single image at the ED phase, with an area under the receiver operating characteristic curve of 0.94 versus 0.58, though the single-image method was significantly faster.

Our work focused on two clinically meaningful phases of the cardiac cycle: end-diastole, when the heart is most relaxed, and end-systole, when it is most contracted. These two extremes capture complementary structural and functional information. We demonstrated that combining ED and ES images improved classification performance compared with using either phase alone, highlighting the diagnostic value of temporal variation in cardiac motion.

Although our results are promising, the limited sample size remains a key constraint—especially for multi-class classification. We achieved a statistically significant improvement through the multi-phase classification model; however, further studies are required to demonstrate its real-time practical utility. Future work will aim to incorporate full cine sequences and larger datasets to further enhance performance and generalizability. Additionally, we plan to explore recurrent or three-dimensional architectures to incorporate full temporal information from cine MRI, extending the capabilities of our multi-phase model.

## 5. Conclusion

Our study demonstrates that a modular, explainable deep learning framework can effectively classify cardiovascular diseases and healthy subjects from cine MRI using only two key cardiac phases: end-diastole and end-systole. The diseases include myocardial infarction, dilated cardiomyopathy, hypertrophic cardiomyopathy and abnormal right ventricle.

In multi-class classification, the ResNet-34 and ResNet-50 models trained on ED and ES frames achieved test F1-scores of 71.0% and 76.0%, respectively. The combined multi-phase model further improved performance to a test F1-score of 77.0%*.* For binary classification, the model achieved even higher F1-scores across disease groups, ranging from 75.0% to 100.0%*.* The use of Smooth Grad-CAM++ enabled the visual explanation of model decisions by highlighting disease-relevant regions in cine MRI, supporting clinical explainability. Despite the relatively small dataset, model performance was robust, aided by careful regularization, transfer learning and cross-validation.

Our findings suggest that explainable deep learning methods trained on clinically relevant cardiac phases can offer valuable, explainable predictions to support clinical decision-making from cine MRI.

## Data Availability

This study uses the publicly available Automated Cardiac Diagnosis Challenge (ACDC) dataset, which can be accessed at https://www.creatis.insa-lyon.fr/Challenge/acdc/databases.html. The code for model training and evaluation is openly available on Zenodo at [26].
